# Bilateral Popliteal Artery Entrapment Syndrome: An Approach to Diagnosis and Salvage

**DOI:** 10.1155/2020/2403280

**Published:** 2020-09-18

**Authors:** Aman Berry Williams

**Affiliations:** The Townsville Hospital and Health Service, Australia

## Abstract

Popliteal artery entrapment syndrome (PAES) is a rare cause of limb-threatening vascular disease. Usually, it arises from aberrant embryological development or acquired dysfunctionality of the popliteal artery and its surrounding musculotendinous structures in the popliteal fossa. Here, we present a case report of a young woman with relatively sudden-onset short-distance claudication and paraesthesia affecting her right leg primarily. She had no recent traumatic history and no atherosclerotic risk factors and was otherwise previously very active. She had a feeble right popliteal artery pulse and no foot pulses. Nerve conduction studies demonstrated no electrophysiological abnormalities. Following computed tomography angiography and magnetic resonance imaging, it was determined she had type 2 PAES. Subsequently, the patient underwent surgical division of a lateralised head of her medial gastrocnemius, resection of her fibrosed popliteal artery, and repair with a reversed long saphenous vein interposition graft. Following surgery, her symptoms resolved, and she remains on aspirin and ultrasound surveillance.

## 1. Full Case Report

A 28-year-old woman was referred by her local general practitioner to the vascular surgical department with intermittent claudication, predominantly affecting her right leg. The claudication had gradually worsened over a 9-month period, now limiting her to a walking distance of 30 metres—relieved by 10-15 minutes of rest. Though denying rest pain, she also described some numbness and pins and needles in her right foot and occasionally had similar sensations in her left. Ultimately, her symptoms were impacting her ability to work, where as a teacher, she found it difficult to walk between classrooms. She denied any previous lower limb trauma, though previously regularly exercised by running. She had no relevant personal or family past medical history, with no significant risk factors for atherosclerotic disease given she was a nonsmoker, nondiabetic, and without hypertension or hypercholesterolemia.

On physical examination, she appeared generally well. A pulse examination demonstrated that she had a feebly palpable right popliteal artery pulse (in comparison to her left) and no palpable pedal pulses. Despite this, she had a normal capillary return, with no lower limb ulceration.

Initially, the patient was referred for Doppler ultrasound, which demonstrated a normal right popliteal vein; however, it did demonstrate a short popliteal artery occlusion with notable large collateral vessels. For completion, blood tests were collected including a vasculitic screen and a thrombophilia screen, both of which were negative. Nerve conduction studies were also then performed, which demonstrated no electrophysiological abnormalities.

**Figure 1:**
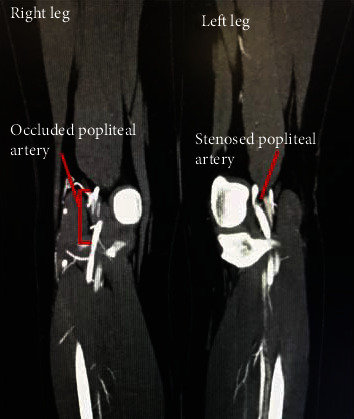
Computed tomography angiography demonstrating a coronal view of the behind knee popliteal arteries, with labelled total occlusion of the right popliteal artery and tight stenosis of the left popliteal artery.

**Figure 2:**
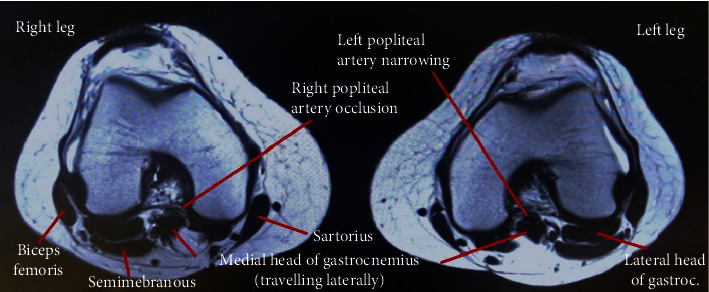
Magnetic resonance imaging demonstrating an axial view of bilateral knees. Note the lateral travel of the medial head of gastrocnemius, resulting in a right popliteal artery occlusion and left popliteal artery high-grade stenosis.

To quantify her disease further, computed tomography angiography (CTA) was conducted confirming in-fact bilateral short-segment popliteal artery disease, with a total occlusion on the right, a high-grade stenosis on the left, and three vessel run-offs bilaterally (Figure 1). Magnetic resonance (MR) angiography was then performed, demonstrating an abnormal medial deviation of bilateral popliteal arteries secondary to an unusually lateral insertion of the medial heads of the gastrocnemius at the superior aspect of the intercondylar notches (Figure 2). Radiographically, this demonstrated a type 2 PAES using the Whelan and Love classification [1]. There was no evidence of popliteal vein entrapment, and the popliteus muscles appeared normal.

**Figure 3:**
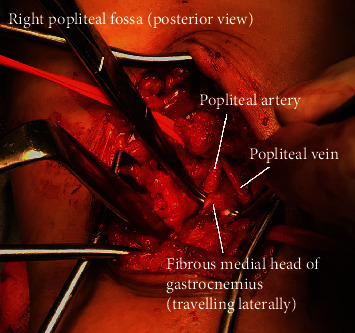
Intraoperative posterior approach of the right popliteal fossa, demonstrating the culprit fibrous muscular band of the medial head of the gastrocnemius.

Following an anaesthetic evaluation, the patient was taken to theatre. Under general anaesthetic, a left leg long saphenous vein harvest was performed, followed by repositioning the patient prone. A lazy-S incision was made over the right popliteal fossa, with appreciation and preservation of the tibial nerve and popliteal vein. The popliteal artery was dissected free, noting a tough muscular band of the medial head of the gastrocnemius travelling laterally over the popliteal artery causing extrinsic compression (Figure 3). This band was divided, and the now released but occluded and fibrosed segment of the popliteal artery was resected. Vessel repair concluded with end-to-end anastomosis of a reversed long saphenous vein interposition graft. Postsurgery, the patient recovered well and after a period of rehabilitation was discharged on aspirin alone. Clinical and ultrasound surveillance monitoring took place at 6 weeks and 3 and 6 months postdischarge, and she now remains on annual graft surveillance. Fortunately, her minor left leg symptoms have completely resolved following participation in a self-motivated claudication walking program.

## 2. Discussion

Popliteal artery entrapment syndrome (PAES) is a rare cause of potentially limb-threatening vascular disease. Typically, it arises from aberrant embryological development or acquired traumatic dysfunctionality of the popliteal artery secondary to surrounding musculotendinous structures in the popliteal fossa [2]. Usually affecting physically active and younger patients (under age 30), common symptoms on presentation include intermittent claudication, paraesthesia, and cold or discoloured peripheries—characteristically relieved by rest [3]. Though usually devoid of other cardiovascular risk factors, as a result of repetitive injury, these patients are prone to further complications such as popliteal artery stenosis, thrombosis, aneurysmal change, and distal lower limb thromboembolic shower—ultimately with potential for acute limb ischaemia [4].

Whether acquired or embryological in nature, given that PAES is generally complex in its development, the potential anatomical variations have been described in a multitude of classification systems—the Whelan and Love system (see [2]), as mentioned earlier, being the most popular. Imaging utility varies greatly with modality. Ultrasound and CTA provide gross arterial perfusion and flow distributions, whereas MR has proved particularly useful in defining extrinsic extra-arterial disease and guiding surgical approach [5]. Furthermore, CTA and MR are particularly pertinent in screening and identifying bilateral disease, where up to 60% of patients with symptomatic PAES will have demonstrable contralateral disease [3].

A high suspicion for PAES should be held when assessing young patients presenting with intermittent claudication, particularly in the absence of other atherosclerotic risk factors. In conjunction with a thorough history and examination, a targeted imaging assessment should be conducted to assist in narrowing the differential diagnosis, as well as prompt for early operative salvage from potential limb-threatening complications.
